# A Novel Protective Framework for Defeating HTTP-Based Denial of Service and Distributed Denial of Service Attacks

**DOI:** 10.1155/2015/238230

**Published:** 2015-05-03

**Authors:** Mohammed A. Saleh, Azizah Abdul Manaf

**Affiliations:** ^1^Faculty of Computing, Universiti Teknologi Malaysia (UTM), 81310 Skudai, Johor, Malaysia; ^2^Advanced Informatics School, Universiti Teknologi Malaysia, 54100 Kuala Lumpur, Malaysia

## Abstract

The growth of web technology has brought convenience to our life, since it has become the most important communication channel. However, now this merit is threatened by complicated network-based attacks, such as denial of service (DoS) and distributed denial of service (DDoS) attacks. Despite many researchers' efforts, no optimal solution that addresses all sorts of HTTP DoS/DDoS attacks is on offer. Therefore, this research aims to fix this gap by designing an alternative solution called a flexible, collaborative, multilayer, DDoS prevention framework (FCMDPF). The innovative design of the FCMDPF framework handles all aspects of HTTP-based DoS/DDoS attacks through the following three subsequent framework's schemes (layers). Firstly, an outer blocking (OB) scheme blocks attacking IP source if it is listed on the black list table. Secondly, the service traceback oriented architecture (STBOA) scheme is to validate whether the incoming request is launched by a human or by an automated tool. Then, it traces back the true attacking IP source. Thirdly, the flexible advanced entropy based (FAEB) scheme is to eliminate high rate DDoS (HR-DDoS) and flash crowd (FC) attacks. Compared to the previous researches, our framework's design provides an efficient protection for web applications against all sorts of DoS/DDoS attacks.

## 1. Introduction

Historically, a series of DDoS attacks that occurred in February 2000 against Amazon, Yahoo, and eBay websites had caused an estimated cumulative loss of 1.2 billion USD. Analysts estimated that during the three hours Yahoo web site was down; it lost about 500,000 USD. According to the bookseller Amazon, the DDoS attack was a reason for losing 600,000 USD during the 10 hours of downtime. Likewise, during the DDoS attacks against eBay, eBay.com availability was degraded from 100% to only 9.4%. In January 2001, Microsoft lost approximately 500 million USD over the course of a few days from a DDoS attack on its site. In 2011, DDoS attacks devastated five high-profile websites, namely, Visa, MasterCard, Sony, WordPress, and the CIA.

Nowadays, DDoS attacks are able to launch a destructive power in a single attack. The biggest peak bandwidth of DDoS in 2010 exceeded 100 Gbps, which represents 100% increase over the size of attack in 2009. As well, peak bandwidth of DDoS in 2013 exceeded 300 Gbps, which represents over three times that of 2010 [1]. Estimates expect that the cost of a 24-hour outage for a large e-commerce company would approach 30 million USD [2, 3].

Denial of service (DoS) attack is an effort by a single machine, namely, an attacker to make a target (server or network) unavailable to its customers, which yields to forbid customers from accessing the service. DoS attack consists of highly damageable attacks to collapse or degrade the quality of service in hardly unexpected manner [4].

Distributed denial of service (DDoS) attack is an attempt to flood a victim, whether is a machine or network, through a volume of traffic that is generated by large number of machines. Furthermore, to diffuse source of attack, these machines are combined from different networks, so it is hard to trace back IP sources of attacks and then to block attacks accordingly [5, 6]. Usually, DDoS attack uses a large number of compromised hosts called zombies or bots that are collected from unprotected computers by planting malicious software on these unprotected computers. Then, these hosts, namely, zombies or bots, are grouped together to shape one huge network called a Botnet, which awaits a command from the attacker to launch the DDoS attack [7–12].

Flash crowd (FC) is a sudden high request in a service caused by legitimate users who simultaneously request the server at the same period. Flash crowd (FC) eventually forces the server to decease its performance and takes it down completely. It occurs due to unexpected big amount of service accesses at the same time. Flash crowd (FC) overwhelms the server, and therefore it causes a denial of service (DoS) attack, which results in either a delay of response or a complete takedown. Flash crowd (FC) could happen due to some exciting event that has just occurred. Likewise, it could be due to the broadcasting of a new service or a free hot software download [2, 13, 14]. From perspectives of service requesters, regardless of whether they are legitimate or illegitimate, flash crowd (FC) may not be counted as an attack. On the contrary, it is counted as an attack from the perspectives of victim or service provider, since it has affected the web server negatively.

Low rate distributed denial of service (LR-DDoS) attack is an intelligent attack that saturates the victim with packets adequately in low rate, in order to avoid the current anomaly-based detection schemes. LR-DDoS attack has an ability to conceal its traffic, since it is identical to normal traffic. LR-DDoS attack is widely used in a large size DDoS attack, which joins several low rate attacks, such as a Botnet to initiate a low rate DDoS attack. LR-DDoS attack produces network traffic similar to the normal network traffic, and, therefore, it is difficult to be detected and mitigated [2, 10, 15].

A high rate distributed denial of service (HR-DDoS) attack is a synonym for the traditional DDoS attacks when attackers exceed and violate the adopted threshold value [15, 16].

Attacker tracing back (TB) can be defined as a method for finding out the exact true IP source of the attacker who launched DoS/DDoS attacks. Client validation (CV) is a method for verifying the validity of the service's requester to validate its legitimacy and illegitimacy and therefore to pass the former and to deny the latter [17].

Outer blocking (OB) is a mechanism for blocking (denying) attackers at the network entrance, more precisely at the Edge Router, which provides network connectivity that is resistant to spoofing attacks. In addition, it helps to save the server's resources, since the attacking IP source is blocked at the outer layer [18].

This research proposed and designed an alternative solution called a flexible, collaborative, multilayer, DDoS prevention framework (FCMDPF), which handles all aspects of HTTP-based DoS/DDoS attacks. FCMDPF framework is flexible because it eliminates the impact of flash crowd (FC) attacks gradually, while it blocks high rate HTTP DoS/DDoS (HR-DDoS) attacks immediately. In addition, it is a collaborative multilayer DDoS prevention framework because it is protecting web server against HTTP DoS/DDoS attacks at different collaborative points through which packets had gone. Each point at different framework's layer collaborates to protect web server from HTTP DoS/DDoS attacks by performing its special tests, and then it forwards the packet to the next framework's layer if it succeeds, or otherwise it will be dropped. In the same manner, the next framework's layer performs its special tests, and then it forwards the packet to the next point if it succeeds, or otherwise it will be dropped, until packet reaches the final target. FCMDPF framework comprises three subsequent multilayer points for detecting and preventing HTTP DoS/DDoS attacks. The first layer of FCMDPF framework is an outer attack blocking (OB) at the edge router, while the second layer of FCMDPF framework is service traceback oriented architecture (STBOA). The third layer of FCMDPF framework is flexible advanced entropy based (FAEB) layer.

The first layer of FCMDPF framework is an outer attack blocking (OB) scheme, which is deployed at the edge router, since it is the most nearest point to the IP attacking source. An outer blocking (OB) scheme first compares and examines the IP source of the incoming request according to its blacklist database table. Then OB scheme blocks or forwards it to the next layer of FCMDPF framework based on whether the incoming request's IP source is listed in blacklist database table at the edge router or not. In case this IP source of the incoming request is not listed on blacklist database table, OB scheme forwards it to the next layer of FCMDPF framework. Otherwise, if it is listed on the blacklist database table, OB scheme blocks it immediately, and host unreachable message will be sent to the requester. This layer provides a helpful service to the web server that all blocking processes will be done at an outer blocking layer, which helps the web server to save its recourses.

An outer attack blocking (OB) scheme is constructed by two integrated components as follows. The first component is blacklist database table, which is used by the OB scheme to record IP sources those are classified as attacking IP sources by STBOA scheme and FAEB scheme in case these IP sources failed to pass their tests. The blacklist database table is created and deployed as well on the edge router, more precisely on Quagga router, which is a part of OB_Shield subsystem. The second component is signaling technique that is used by STBOA scheme and FAEB scheme to report attacking IP sources to OB scheme and therefore to update its blacklist database table and hence to block these IP sources on upcoming requests.

The second layer of FCMDPF framework is service traceback oriented architecture (STBOA) scheme that is designed to validate whether the incoming request is launched by a human (real web browser) or by an automated tool (bots). Then, it traces back the incoming request in order to find out the true IP attacking source. Service traceback oriented architecture (STBOA) scheme is designed based on service traceback oriented architecture (STBOA) algorithm.

The third layer of FCMDPF framework is flexible advanced entropy based (FAEB) scheme, which is employed to detect anomalies in HTTP network traffic and to differentiate whether it is high rate DDoS (HR-DDoS) attacks or flash crowd (FC) attacks. Flexible advanced entropy based (FAEB) scheme is designed based on flexible advanced entropy based (FAEB) algorithm. In case FAEB classifies that the incoming HTTP network traffic is high rate HTTP DoS/DDoS (HR-DDoS) attacks, it blocks it immediately. Whereas if FAEB classifies that the incoming HTTP network traffic is flash crowd (FC) attacks, it decreases the maximum connection's timeout value and decreases the maximum allowed request per this timeout, until these two values reach zero. Once the values of timeout and the maximum allowed requests reach zero, FAEB scheme disables KeepAlive feature of HTTP connection. Therefore, the mode is exchanged from detecting and preventing flash crowd attack to detecting and preventing high rate DDoS attack. In addition, FAEB scheme uses signaling technique to update the edge router's blacklist database.

Moreover, Section 3 in this paper provides full and granular details of FCMDPF framework. Lastly, FCMDPF framework is evaluated based on the analysis of experimental simulations, as is described in Section 4.

This paper is organized as follows. First, Section 1 introduced the interested topic, defined the relevant terms, and provided high-level description of FCMDPF framework. Then, Section 2 reviewed the previous related works. It classified existing frameworks and schemes that protect web applications from HTTP-based DoS and DDoS attacks, conducted survey on them, and identified the optimal specifications that should be offered by a protective framework to protect web applications from all sorts of HTTP-based DoS and DDoS attacks. After that, Section 3 provided full and granular details, or low-level description, of FCMDPF framework. As well, it explained the systematic procedures of the framework's evaluation. Next, Section 4 presented discussion and analysis. Finally, Section 5 concluded this paper.

## 2. Literature Review

This section identifies problems in the current related works and also describes the optimal framework specifications. The literature review began by classifying existing schemes and frameworks into different categories. Secondly, it conducted a comprehensive survey of detection and prevention schemes and frameworks for all sorts of HTTP-based DoS and DDoS attacks in order to show the problems with each related work. Finally, it described the optimal specifications for a protective framework against HTTP-based DoS and DDoS attacks, which fix all the shortcomings found in previous related works, as are set out below.

### 2.1. Classifying Existing Schemes and Frameworks

Based on extensive studies and analysis of the related works, existing schemes and frameworks can be classified into one or more of five categories. The five categories are high rate DDoS (HR-DDoS) attacks, low rate DDoS (LR-DDoS) attacks, flash crowd (FC) attacks, outer blocking (OB), and traceback and client validation (TB and CV). Various researchers [1, 19–23] highlighted that the protective scheme or framework should protect web applications from high rate DDoS (HR-DDoS) attacks, whilst other researchers suggested it should provide a protection for web applications from Low Rate DDoS (LR-DDoS) attacks [24, 26]. Other researchers [26, 27] claimed that it should provide protection against flash crowd (FC) attacks. Likewise, another group [20, 28, 29] emphasized it should be able to trace back (TB) to the true source of the attack, verify the client's validity (CV), and block it at the edge router as well (OB).

### 2.2. Survey on the Detective and Protective Schemes and Frameworks That Protect Web Applications from HTTP-Based DoS and DDoS Attacks

Table 1 presents a comprehensive survey of the detective and preventive schemes and frameworks that handles all sorts of HTTP-based DoS and DDoS attacks. The survey is conducted according to the five categories that are identified in the previous subsection.

### 2.3. Optimal Specifications for Detective and Protective Framework to Protect Web Applications from All Sorts of HTTP-Based DoS and DDoS Attacks

Based on the survey unveiled in Table 1 above and a review of the related works, the optimal specifications for a protective framework against HTTP-based DoS and DDoS attacks are required to provide full support to all of the following features.

1. The framework should provide a protection against both HR-DDoS and FC attacks. Due to similarities between HR-DDoS and FC attacks, the framework should be able to differentiate between them clearly to block the former immediately and block the latter gradually.
2. The framework should provide a protection against LR-DDoS attacks.
3. The framework should provide a mechanism to verify the validity of the incoming requests whether they are legitimate (normal web browser) or illegitimate (botnet). In addition, the mechanism should be able to pass the former and block the latter. As well, the framework should provide a mechanism to find out the true attacking IP source. The mechanism here should not be designed in a way that annoys the requesters (clients) by performing extra tasks such as CAPTCHA.
4. The framework should provide a mechanism to block the attacking IP sources at the edge router (network entrance) near to the attacking source. The benefit of this technique helps to save the resources of web servers, since the blocking occurs before incoming requests reach the web server.
5. The framework should be designed in a way that supports the concept of separation of duties in order to prevent a single point of failure problem. The framework's components should be deployed on different layers and collaboratively work together to protect web applications from HTTP-based DoS and DDoS attacks.
6. The framework should be compatible with existing protocols.
7. The framework should be designed explicitly for processing the web application layer; HTTP protocol, rather than only the network layer; IP and ICMP protocols or the transport layer; TCP and UDP protocols.
8. The framework should be easy to implement without causing processing and bandwidth overheads.
9. The framework design should be dynamically able to adopt and update itself, once needed.
10. The framework design should provide support to the hybrid scheme, which comprises the proactive and reactive schemes. The proactive scheme is required for client validation and traceback (CV and TB), whilst the reactive scheme is required for protecting against high rate DDoS (HR-DDoS) and flash crowd (FC) attacks.
11. The framework should consume low storage memory.
12. The framework should be resistant to IP source spoofing attacks, especially when finding out the true attacking IP sources.

## 3. Flexible, Collaborative, Multilayer, DDoS Prevention Framework (FCMDPF)

### 3.1. Design of FCMDPF Framework

As is shown previously in the literature review, all of the related works failed or at least could not protect web applications from HTTP DoS/DDoS attacks properly. The DoS/DDoS attacks are varying from high rate DoS/DDoS (HR-DDoS) attacks, low rate DoS/DDoS (LR-DDoS) attacks, and flash crowd (FC) attacks. The perfect protective solution should provide a protection from all of these mentioned attacks. In addition, it should be able to trace back attacking IP sources of the DoS/DDoS attacks in order to block them. From there, this research paper proposed and designed a comprehensive protective solution that handles all sorts of HTTP DoS/DDoS attacks called a flexible, collaborative multilayer, DDoS prevention framework (FCMDPF).

The FCMDPF framework is flexible because it eliminates the impact of flash crowd (FC) attacks gradually by decreasing the maximum connection's timeout value and decreasing the maximum allowed request per this timeout, until these two values reach zero. Once the values of timeout and the maximum allowed requests reach zero, FAEB scheme disables KeepAlive feature of HTTP connection. Therefore, the mode is exchanged from detecting and preventing flash crowd attack to detecting and preventing high rate DDoS attack. In the meanwhile, FCMDPF framework blocks high rate HTTP DoS/DDoS attacks immediately. In addition, it is a collaborative multilayer DDoS prevention framework because it protects web server against HTTP DoS/DDoS attacks at different collaborative points through which packets had gone. Each point at different framework's layer collaborates to protect web server from HTTP DoS/DDoS attacks by performing its special tests and then it forwards the packet to the next framework's layer (point) if it succeeds, or otherwise it will be dropped. In the same manner, the next framework's layer performs its special tests, and then it forwards the packet to the next point if it succeeds, or otherwise it will be dropped, until packet reaches the final target, which is the web application.

**Figure 1:**
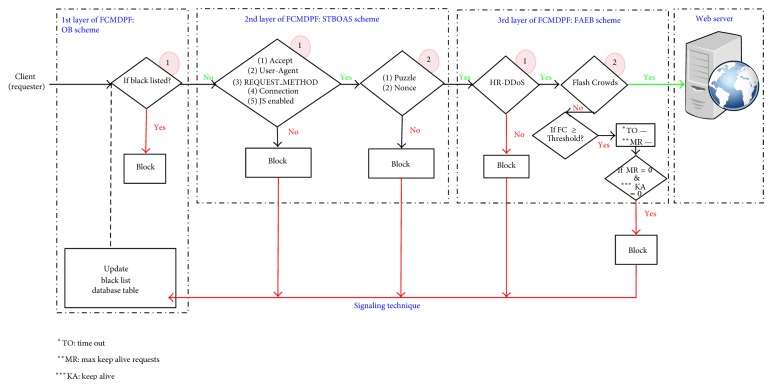
Components of flexible collaborative multilayer DDoS prevention framework (FCMDPF).

The FCMDPF framework comprises three subsequent multilayer points for detecting and preventing HTTP DoS/DDoS attacks. The first layer of FCMDPF framework is an outer attack blocking (OB) at the edge router while the second layer of FCMDPF framework is service traceback oriented architecture (STBOA). The third layer of FCMDPF framework is flexible advanced entropy based (FAEB) layer.  Figure 1 illustrates the components of flexible collaborative multilayer DDoS prevention (FCMDPF) framework. As well, a protective system, namely, AntiDDoS_Shield, is developed based on FCMDPF framework.

The first layer of FCMDPF framework is an outer attack blocking (OB) scheme, which is deployed at the edge router, since it is the nearest point to the attacking IP source. An outer blocking (OB) scheme first compares and examines the IP source of the incoming request according to its blacklist database table. Then, OB scheme blocks or forwards it to the next layer of FCMDPF framework based on whether the incoming request's IP is listed in blacklist database table at the edge router or not [26–30]. In case this IP source of the incoming request is not listed on blacklist database table, OB scheme forwards it the next layer of FCMDPF framework, but if it is listed on the blacklist database table, OB scheme blocks it immediately and host unreachable message will be sent to the requester. This layer provides a helpful service to the web server, since all blocking processes will be done at an outer blocking layer, which helps the web server to save its recourses [6, 11, 21]. In addition, this research developed protective subsystem called OB_Shield, which is part of AntiDDoS_Shield system, based on OB scheme. OB_Shield subsystem employed ready configured Quagga and iproute2 routing suites. In addition, it integrated signaling technique that is used by STBOA_Shield subsystem and mod_antiddos subsystem to report back attacking IP sources to OB_Shield subsystem in order to update its blacklist database table.

An outer attack blocking (OB) scheme is constructed by two integrated components as follows. The first component is blacklist database table, which is used by the OB scheme to record IP sources that are classified as attacking IP sources by STBOA scheme or FAEB scheme in case if these IP sources failed to pass the tests of STBOA scheme or FAEB scheme. The blacklist database table is created and deployed as well on the edge router, Quagga router, which is a part of OB_Shield subsystem. The second component is signaling technique that is used by STBOA scheme and FAEB scheme to report attacking IP sources to OB scheme. Therefore, STBOA scheme and FAEB scheme update OB scheme's blacklist database table and hence block these IP sources on upcoming requests.

In fact, the outer blocking (OB) scheme, that is deployed at the edge router, besides using of signaling technique, is a new novel scheme in reference to the previous related works. The edge router's blacklist database will be updated with new IP sources in case the incoming request failed to satisfy STBOA scheme or FAEB scheme. The updating of new IP source leads to judging that the incoming IP source is an attacker. Then, the FCMDPF framework uses signaling technique to classify the attacking IP source, so that IP source will be traced back, black listed, and blocked, as well.

**Figure 2:**
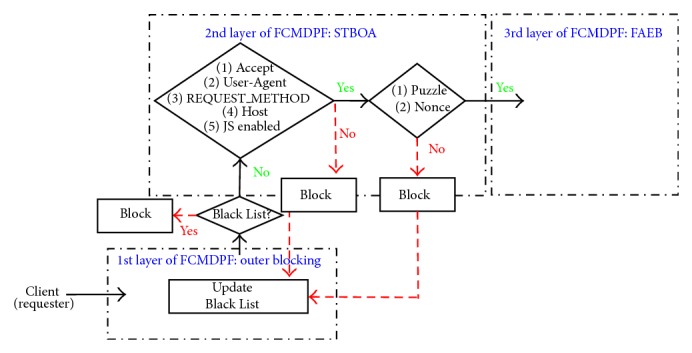
STBOA scheme processes and validates the incoming requests.

The second layer of FCMDPF framework is service traceback oriented architecture (STBOA) scheme that is designed to validate whether the incoming request is launched by a human (real web browser) or by an automated tool (bots). Then, it traces back the incoming request in order to find out the true attacking IP source. Service traceback oriented architecture (STBOA) scheme is designed based on service traceback oriented architecture (STBOA) algorithm, as it is shown in Algorithm 1.  Figure 2 demonstrates how STBOA scheme processes, treats, and validates the incoming requests. In addition, this research developed subsystem called STBOA_Shield, which is part of AntiDDoS_Shield system, based on STBOA scheme in order to validate and trace back the attacking source. STBOA_Shield subsystem is a web application that is developed by using SOAP and PHP scripting programming language.

First off, service traceback oriented architecture (STBOA) scheme validates the incoming request to determine whether the request is legitimate, which is launched by legal user, or illegitimate one that is executed by an automated tool, such as a bot. The purpose of this process is to identify illegitimate requests that are launched by IRC bots in order to block them immediately. After that, STBOA scheme traces back the incoming request in order to find out the true attacking IP source. STBOA scheme accomplishes these missions through different two subsequent stages.

In the first stage, STBOA scheme validates the incoming request by checking the request's header looking for unique header's values, which are carried out only by legitimate request, such as web browsers [31–37].  Algorithm 2 presents the unique header's values that are checked by STBOA scheme. STBOA scheme checks for “User-Agent”, “Accept”, and “Host” headers in HTTP connection, and it makes sure that the requester (client) has enabled its own Javascript language engine. Furthermore, it checks for “REQUEST_METHOD” header's value whether it is GET, HEAD, or POST [31, 38–44]. If the incoming request passes all of these checks, it will proceed to the next test. Otherwise, it will be terminated and blocked immediately, and the signal is sent to the edge router through signaling technique, so that the client will be blocked at the edge router.

**Figure 3:**
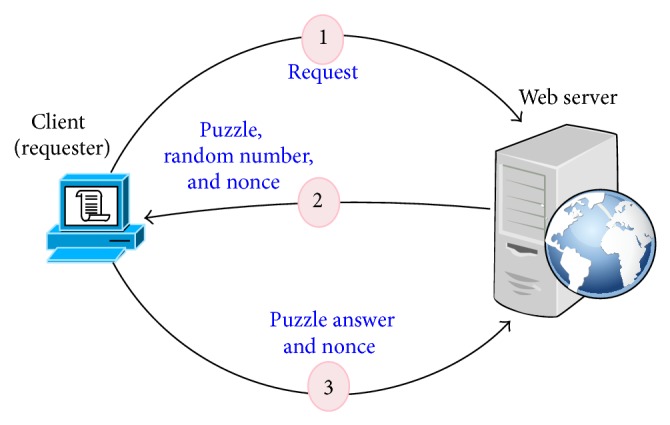
Conceptual steps of the STBOA web service puzzle for validating clients.

In the second stage, STBOA scheme utilizes web service technology to formulate and generate a puzzle, random number, and nonce value. Then, it sends them back to the client or the requester [32–37, 45–49]. The client has to solve a puzzle by using a random number that is sent by web server (web application). Then, the client sends back the solved puzzle (puzzle's answer), along with the nonce value. After that, the web server (web application) will verify puzzle's answer and nonce value that are sent by the client whether they are correct or not. If both numbers are correct, the request will be forwarded for the next test. Otherwise it will be blocked immediately and a signal is sent back to the edge router to update its black list.  Figure 3 demonstrates how STBOA scheme utilizes web service technology to validate a client.

In this research paper, formulating and generating a puzzle and nonce value are done based on web service by STBOA scheme. It is the most appropriate and preferred solution compared with the other similar solutions, such as CAPTCHA, which annoys the clients [13]. It is preferred because it does not burden a client to solve a puzzle and send the answer back to web server, since a legitimate web browser does this mission without client interception. In addition, the nonce value plays a significant role, since it is used for an extra verification purpose to ensure that the right client answered the puzzle. STBOA scheme formulates and generates a puzzle, random number, and nonce value based on formulas in Algorithm 3.

Service traceback oriented architecture (STBOA) scheme, that is deployed in the second layer of FCMDPF framework, is an expansion and modification to the previous works done by Subbulakshmi et al. [1], Yang et al. [7], Mohan and Raji Reddy [12], Wang et al. [21], and Darapureddi et al. [22] in order to validate whether the request is launched by a human or an automated tool, such as a bot. STBOA scheme adopted and employed extra specifications such as supporting cookie by the client, calculating, solving some random puzzles, and sending valid requests to ensure the request launched by a human.

**Figure 4:**
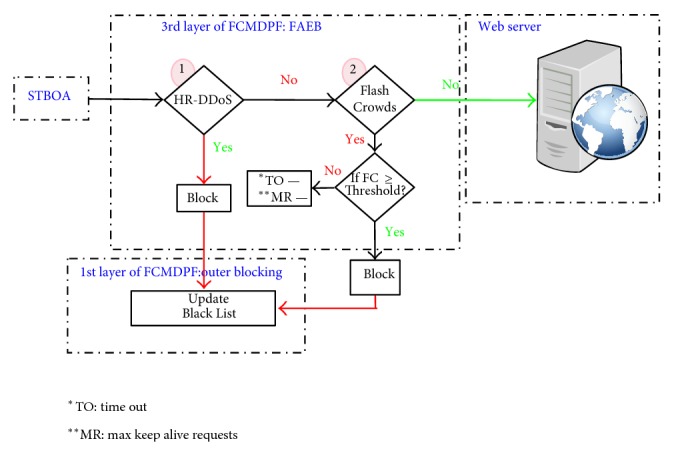
FAEB scheme verifies the incoming requests.

The third layer of FCMDPF framework is flexible advanced entropy based (FAEB) scheme, which is employed to detect anomalies in HTTP network traffic and to differentiate whether it is high rate DDoS (HR-DDoS) attack or flash crowd (FC) attack. Flexible advanced entropy based (FAEB) scheme is designed based on flexible advanced entropy based (FAEB) algorithm, as it is shown in Algorithm 4. In case FAEB scheme classifies that the incoming HTTP network traffic is high rate HTTP DoS/DDoS (HR-DDoS) attack, it blocks it immediately, whereas if FAEB scheme classifies that the incoming HTTP network traffic is flash crowd (FC) attack, it decreases the maximum connection's timeout value and it decreases the maximum allowed request per this timeout, until these two values reach zero. Once the values of timeout and the maximum allowed requests reach zero, FAEB scheme disables KeepAlive feature of HTTP connection. Therefore, the mode is exchanged from detecting and preventing flash crowd attack to detecting and preventing high rate DDoS attack. In addition, FAEB scheme uses signaling technique to update the edge router's blacklist database.  Figure 4 demonstrates how FAEB scheme processes, treats, and verifies the incoming requests.

First off, FAEB scheme examines the incoming request to determine whether the request belongs to white list table or blacklist table of Apache web server, as it is explained in Algorithm 5. In the former case, if the IP source of the incoming request belongs to IP sources in white list, it will be excluded from checking, and it always be allowed for accessing the web server (web application), while in the latter case, if the IP source of the incoming request belongs to IP sources in black list, it will be blocked immediately, and a signal is sent to update blacklist database table of the edge router.

After that, FAEB scheme examines an Apache web server to figure out whether it is under high rate DDoS attack and flash crowd attack or in normal situation. FAEB scheme does so periodically based on the adopted time in the module's configuration by calculating entropy of overall requests through the following formulas [50–55]:

(1) $$\begin{array}{c} \text{entropy}=\text{Pi}\;*\log_{2}\text{Pi},\\ \text{Pi}=\frac{\text{uri}\_\text{counts}}{\text{total}\_\text{counts}}. \end{array}$$

Then, FAEB scheme compares the computed result of entropy with the thresholds value of high rate DDoS (HR-DDoS) and flash crowds (FC) attacks that are adopted during the system's profiling. If FAEB scheme determines that Apache web server is under high rate DDoS attacks, it then blocks all requests that shared and participated in attacks, and it reports them to the edge router in order to update its black list, while if FAEB scheme determines that an Apache web server is under flash crowds attack, it decreases the maximum connection's timeout value and it decreases the maximum allowed request per this timeout, until these two values reach zero. Once the values of timeout and the maximum allowed requests reach zero, FAEB scheme disables KeepAlive feature of HTTP connection. Therefore, the mode is exchanged from detecting and preventing flash crowd attack to detecting and preventing high rate DDoS attack. Once the calculated entropy exceeds the maximum threshold's value of flash crowd attack, it then blocks all incoming requests that participated in attack. Then, it reports them to the edge router in order to update its black list through signaling technique. Whereas the Apache web server is neither under high rate DDoS, nor under flash crowds, it is considered under normal situation. Therefore, the incoming requests are treated as legitimate requests.

The FAEB scheme detects and prevents flash crowd (FC) attack by calculating the entropy of incoming requests that are launched towards hot pages of the website. The reason behind choosing hot web pages to simulate the flash crowd (FC) attack is that the legitimate users suddenly launch a large number of requests towards the web application. Indeed, these requests eventually overwhelm the server, and, therefore, they cause a denial of service (DoS) attack, which results in either a delay of response or a complete takedown [2, 13, 14, 29, 38]. The FAEB scheme detects and prevents flash crowd (FC) attacks by calculating the entropy based on the flash crowd attack entropy algorithm, as is shown in Algorithm 6.

**Figure 5:**
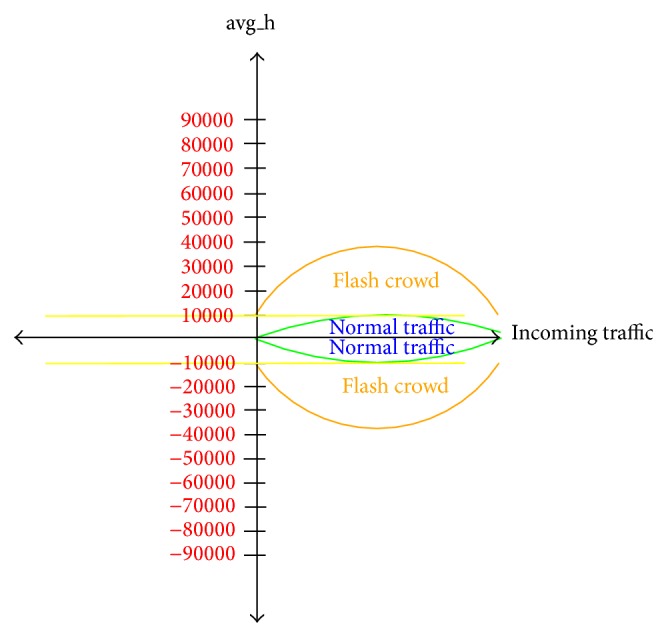
Clicks' average of the hot web pages.

The flash crowd (FC) attack entropy algorithm first calculates clicks' average of the hot web pages, and if it exceeds 10000, as depicted in Figure 5, it starts to calculate the entropy [43]. The reason behind 10000 clicks' average on web pages condition is that the entropy compares the calculated value to threshold, which is classified as long-term entropy based on [23]. If the calculated entropy is outside the range −0.5 <* H* < +0.5, it indicates that a flash crowd (FC) attack is taking place. Otherwise, it is not flash crowd (FC) attack.

Likewise, the FAEB scheme detects and prevents the HR-DDoS attack by calculating the entropy of incoming requests that are launched towards cold pages of a website [2, 13, 14, 29, 38]. The FAEB scheme detects and prevents the HR-DDoS attack by calculating the entropy of incoming requests based on the high rate attack entropy algorithm, as is shown in Algorithm 7.

**Figure 6:**
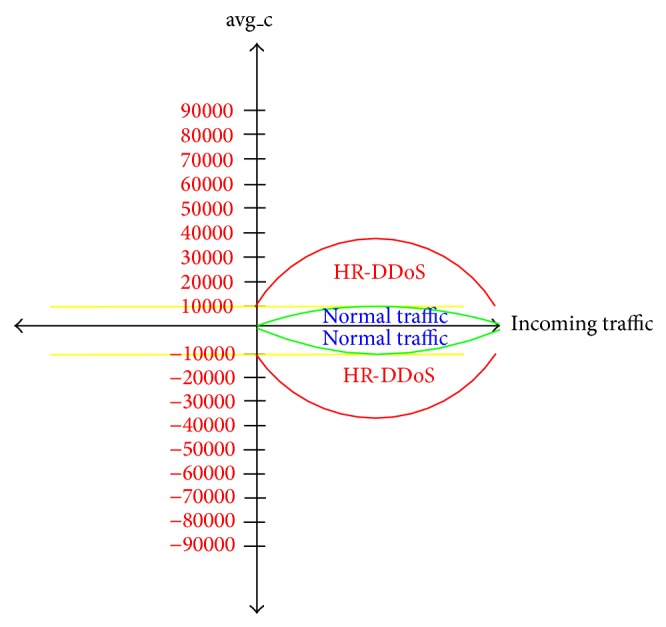
Clicks' average of the cold web pages.

The high rate attack entropy algorithm first calculates the clicks' average for the cold web pages, and if it exceeds 10000, as shown in Figure 6, it starts to calculate the entropy [43]. The reason behind 10000 clicks' average on web pages condition is that the entropy compares the calculated value to threshold, which is classified as long-term entropy based on [23]. If the calculated entropy is outside the range −1.36 <* H* < +1.36, it indicates that a HR-DDoS attack is taking place. Otherwise, it is not high rate attack.

Finally, the FAEB scheme blocks incoming requests that represents high rate DDoS attack (HR-DDoS) immediately, while it blocks incoming requests that represents a flash crowd (FC) attack gradually by decreasing the maximum connection's timeout value and decreasing the maximum allowed request per this timeout, until these two values reach zero. Once the values of timeout and the maximum allowed requests reach zero, FAEB scheme disables KeepAlive feature of HTTP connection. Therefore, the mode is exchanged from detecting and preventing flash crowd (FC) attack to detecting and preventing high rate DDoS (HRDDoS) attack. In addition, the FAEB scheme feeds back those blocked IP sources to outer attack blocking (OB) scheme's blacklist database table through signaling technique. Otherwise, the FAEB scheme passes incoming requests that represent a normal traffic. Besides that, this research developed subsystem called mod_antiddos Apache module, which is part of AntiDDoS_Shield system, based on FAEB scheme. The mod_antiddos subsystem is an Apache web server module, which is programmed by using Apache APR library and C programming language.

**Figure 7:**
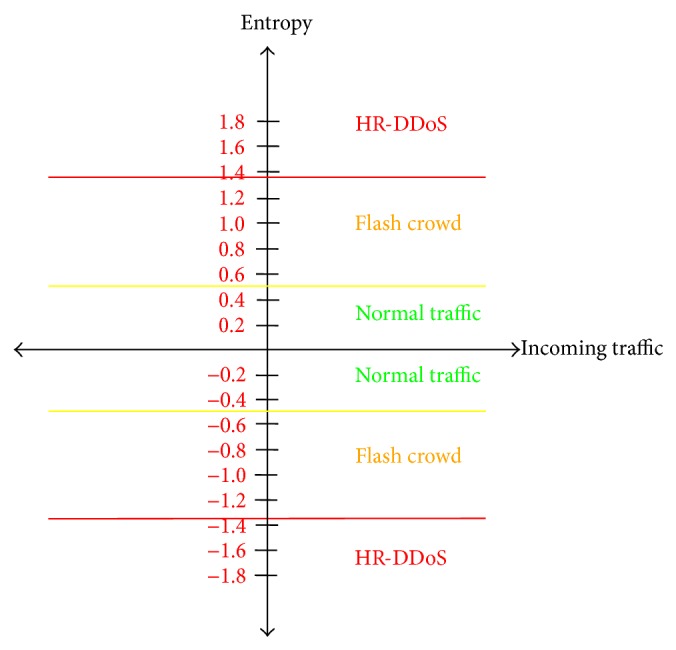
The overall entropy for the flash crowd (FC) attack case, high rate DDoS (HR-DDoS) attack case, and a normal traffic case.

Figure 7 presents the overall entropy for the three different cases: flash crowd attack case, high rate DDoS attack case, and normal traffic case.

Flexible advanced entropy based (FAEB) scheme, that is deployed in third layer of FCMDPF framework, is an expansion to the previous related works done by Chonka et al. [6], Xie and Tang [10], (Zheng et al. [18]), Wen et al. [20], Schweizer [56], Ciufo [57], Monshouwer [58], Ye and Zheng [13], Sqalli et al. [8], and SpiderLabs ModSecurity [59]. FAEB scheme provides an ideal protective solution for high rate HTTP DoS/DDoS (HR-DDoS) and flash crowd (FC) attacks smoothly by blocking high rate DoS/DDoS attacks immediately, while blocking flash crowd attacks gradually. On the other hand, offering a protective solution against the low rate HTTP DoS/DDoS (LR-DDoS) attacks is ignored intentionally in this research, since such a protection now is available by default in all recent web servers. Despite this fact, FAEB is able to provide a protection against the low rate HTTP DoS/DDoS (LR-DDoS) attacks.

### 3.2. Evaluating FCMDPF Framework

In this research paper, evaluating FCMDPF framework is done based on simulation of practical experiments of the AntiDDoS_Shield system, which is developed based on FCMDPF Framework, and the analysis of corresponding experimental results. As is explained earlier, the AntiDDoS_Shield system is developed in this research, as well. In fact, four different types of experiments are launched for testing and evaluating the AntiDDoS_Shield system. The first type of experiment is to test and evaluate the work for providing a protection against flash crowd (FC) attacks, while the second type of experiment is to test and evaluate the work for providing a protection against high rate DDoS (HR-DDoS) attacks. The third type of experiment is to test and evaluate the work for validating the client and tracing back the true IP source of attack. The fourth type of experiment is to test and evaluate the concerned work for blocking attacking IP source, as nearest as possible to attacking IP source, at the edge router, network entrance.

Simulation of flash crowd (FC) attack's experiment is accomplished by launching huge number of distributed incoming requests against hot pages of the website (hot web pages). Several studies proved that hot web pages represent about 10 percent of whole web pages [14, 16]. The reason behind choosing hot pages to simulate flash crowd (FC) attack is that the legitimate users launch sudden high requests for accessing them. It eventually overwhelms the server and, therefore, causes a denial of service (DoS) attack, which results in either a delay of response or a complete takedown [4, 60, 61], while, simulating high rate (HR-DDoS) DDoS attack's experiment is carried out by launching large number of distributed requests against cold pages of website, whereas the third and fourth experiments are accomplished by simulating incoming legitimate and illegitimate requests towards an Apache web server.

**Figure 8:**
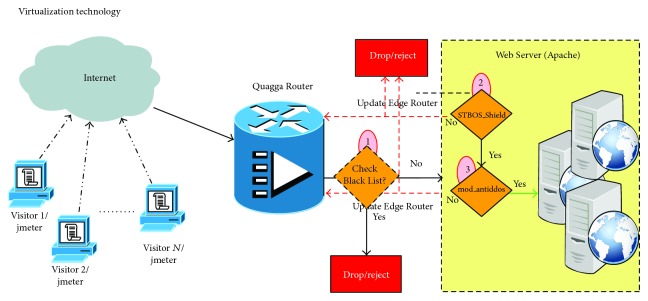
The construction simulation environment.

The simulation environment is constructed by using virtualization technology to include all of the needed vectors and players. As is explained in Figure 8 from right to left, it consists of:

- The Apache web server serves the incoming requests and responds to them accordingly. Two of developed subsystems, namely, STBOA_Shield subsystem and mod_antiddos subsystem, are installed on Apache and are configured, as well.
- The Quagga and iproute2 routing suites software are employed on the edge router at the entrance of the network. The main objective of these two tools is to permit or deny network traffic routing to inside and outside of the network.
- There are web application legitimate clients (customers) and attackers.

The ways to know and therefore to detect whether there is an attack or not are through one of the following ways.

- The incoming request fails to pass the validation tests successfully. The validation's tests require that the incoming request carries out on its HTTP header all of the following pair's values: User-Agent, Accept, Host, and REQUEST_METHOD, and Javascript language engine is enabled. As well, if the incoming request fails to answer the generated puzzle successfully, it is flagged as an attack.
- The attacker launches huge volume of incoming requests that are headed against the hot web pages of the web application, which cause flash crowd (FC) attack.
- The attacker launches huge volume of incoming requests that are headed against the cold web pages of the web application, which cause high rate DDoS (HR-DDoS) attack.
- The edge router at the network entrance fails to inspect the incoming requests. Therefore, it fails to detect and prevent the attacking IP sources at this point before the incoming request traverses or move to the inside (private) network.

**Figure 9:**
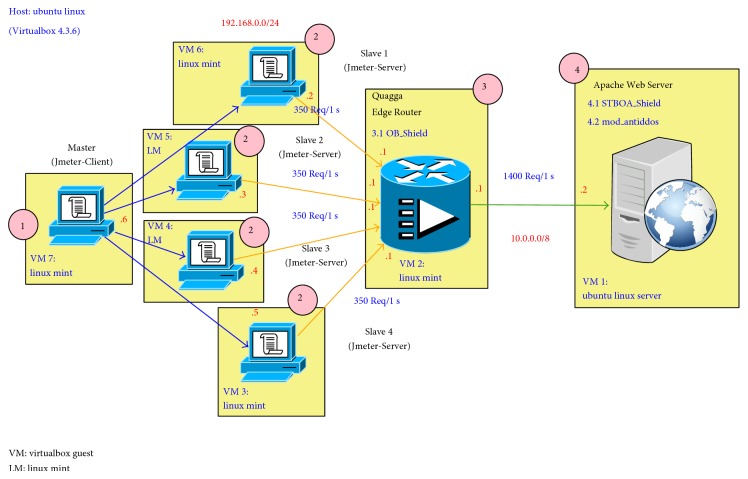
Sequential steps of simulation model.

The simulation model, as is shown in Figure 9, is conducted for each one of the four different simulations through the five following steps.

1. Jmeter-Client opens the corresponding distributed testing plan, one plan out of four plans that are listed below for each simulation, and then it sends a command to Jmeter-Servers:
  - client validation and traceback testing Plan.jmx;
  - flash crowd attack testing Plan.jmx;
  - high rate DDoS attack Plan.jmx;
  - edge router outer blocking testing Plan.jmx.
2. Jmeter-Servers simulate the required incoming requests based on the distributed testing plan, which is sent by Jmeter-Client.
3. The OB_Shield subsystem, Quagga Router, first checks and examines the IP source of the incoming request based on its blacklist database table. If this IP source is listed on the black list database table, OB_Shield blocks it immediately by responding back to the requester with host unreachable message. Otherwise, it forwards it to Apache web server.
4. The STBOA_Shield subsystem validates the incoming request to ensure that is launched by a human not by an automated tool like botnet. If this incoming request succeeds to pass this test, which is launched by a human, it will proceed to the next test of STBOA_Shield subsystem. Otherwise, STBOA_Shield subsystem blocks it immediately by responding back to the requester with HTTP_FORBIDDEN message. Then, STBOA_Shield subsystem sends back a puzzle to the requester, which the requester needs to solve it correctly. If the requester passes this test too, it will proceed to the next test of mod_anitddos subsystem. Otherwise, STBOA_Shield subsystem blocks it immediately by responding back to the requester with HTTP_FORBIDDEN message. As well, when STBOA_Shield subsystem responds to the requester with HTTP_FORBIDDEN message, it reports the attacking IP sources to OB_Shield subsystem in order to update its blacklist database table through signaling technique.
5. The mod_antiddos subsystem checks Apache web server to determine whether it is under high rate DDoS attack and flash crowd attack or in normal situation. If Apache is under high rate DDoS attack, the mod_antiddos subsystem blocks incoming requests immediately by responding back to the requesters with HTTP_FORBIDDEN message. While if it is under flash crowd attack, the mod_antiddos subsystem blocks incoming requests gradually by decreasing the maximum connection's timeout value and decreasing the maximum allowed request per this timeout, until these two values reach zero. Once the values of timeout and the maximum allowed requests reach zero, FAEB scheme disables KeepAlive feature of HTTP connection. Therefore, the mode is exchanged from detecting and preventing flash crowd (FC) attack to detecting and preventing high rate DDoS (HRDDoS) attack. In the meanwhile, FCMDPF framework blocks high rate HTTP DoS/DDoS attacks immediately. Otherwise, the incoming request accesses its final target. As well, when the mod_antiddos subsystem responds to the requester with HTTP_FORBIDDEN message, it reports the attacking IP sources to OB_Shield subsystem in order to update its blacklist database table through signaling technique.

The parameters or variables that are measured during the simulation of the four experiments for testing and evaluating the work are as follows.

1. There is the quantity of incoming requests that are simulated by Jmeter-Servers in each one out of the four different simulations.
2. The quantity of the detected and prevented attacks that are launched against the web application: in the first three simulations, the quantity of the detected and prevented attacks is gathered from Apache log file for the work. The work should respond to the incoming request with HTTP response code number 403 (forbidden), 500 (internal server error), or 503 (service unavailable), depending on the behaviour of the work. On the contrary, once the work responds to the incoming request with HTTP response code number 200, it indicates that the work failed to detect and prevent the attack, while in the last simulation, the quantity of the detected and prevented attacks is gathered from network traffic that is captured and saved in.pcap format for the work. Once the work detects and prevents the attack, it responds to the requester with host unreachable message or otherwise the work failed to detect and prevent the attack.

The actual simulation model generated 420000 incoming requests for each simulation because it is the minimum required number that causes DoS/DDoS attack [2, 20, 23]. Therefore, it generated 420000 incoming requests in order to test and evaluate blocking and tracing back the attacking IP sources at the edge router. As well, it generated 420000 incoming requests in order to test and evaluate validation of the incoming requests. Besides that, it generated 420000 incoming requests in order to test and evaluate detecting and preventing high rate HTTP DoS/DDoS (HR-DDoS) attack. In addition, it generated 420000 incoming requests in order to test and evaluate detecting and preventing flash crowd (FC) attack.

The AntiDDoS_Shield system detected and prevented all high rate HTTP-based DoS/DDoS (HR-DDoS) attacks, which were 420000 high rate HTTP-based DoS/DDoS attacks, through mod_antiddos module subsystem. The mod_antiddos subsystem first calculated clicks' average of the cold web pages that exceeded 10000. Then, it calculated the entropy, which was out of −1.36 < *H* < +1.36 range. Hence, the mod_antiddos subsystem indicated that the web server is under high rate DDoS attack. Therefore, it blocked all incoming requests immediately by replying to them with HTTP_FORBIDDEN message or HTTP response code number 403. Then, it updated OB_Shield with these attacking IP sources, as well.

In addition, the AntiDDoS_Shield system detected and detected and prevented 369726 out of 420000 flash crowd (FC) attacks through mod_antiddos module subsystem. The mod_antiddos subsystem first calculated clicks' average of the hot web pages that exceeded 10000. After that, it calculated the entropy, which was out of −0.5 < H < +0.5 range. Hence, the mod_antiddos subsystem indicated that the web server is under flash crowd (FC) attack. Therefore, it blocked all incoming requests gradually by decreasing the maximum connection's timeout value and decreasing the maximum allowed request per this timeout to the half, until these two values reached zero. Then, the mod_antiddos subsystem disabled KeepAlive feature of HTTP connection, and, therefore, the detection and prevention mode is exchanged from flash crowd attack to high rate DDoS attack. Thus, the mod_antiddos subsystem blocked flash crowd attacks gradually by replying to them with HTTP_FORBIDDEN message or HTTP response code number 403. Lastly, it updated OB_Shield with these attacking IP sources, as well.

As well, the AntiDDoS_Shield system succeeded to detect and prevent all of those attacking IP sources, which were 420000 IP sources of incoming requests, at the edge router, namely, Quagga edge router by replying to the requester with host unreachable message.

Besides that, AntiDDoS_Shield system succeeded to validate and trace back 369726 out of 420000 incoming requests by STBOA_Shield subsystem. It validated incoming requests that are missing User-Agent header, Accept header, Host header, REQUEST_METHOD header, or disabled Javascript language engine. Besides that, it traced back incoming requests through a puzzle.

## 4. Discussion and Analysis 

Finally, this section discusses and evaluates our proposed and designed protective framework for defeating HTTP-based DoS/DDoS attacks, namely, the flexible, collaborative, multilayer, DDoS prevention framework (FCMDPF). The FCMDPF framework is evaluated based on the optimal specifications for a protective framework to protect web applications from all sorts of HTTP-based DoS and DDoS attacks that are outlined above.  Table 2 provides all evaluation details.

## 5. Conclusions

Although many researchers focused on proposing and designing robust schemes and frameworks for protecting web applications from all sorts of HTTP-based DoS/DDoS attacks, there are still open issues that need to be addressed, as are described previously in the literature review. This research paper proposes and designs a novel protective framework for defeating HTTP-based DoS/DDoS attacks, namely, the flexible, collaborative, multilayer, DDoS prevention framework (FCMDPF). The novelty of this framework's design fixes and overcomes all the shortcomings of the previous related works. It provides a novel alternative protective framework to protect web applications from all sorts of HTTP DoS/DDoS attacks, such as high rate DDoS (HR-DDoS) and flash crowd (FC). In addition, it is quite able to validate and trace back (TB and CV) the real attacking IP sources and block them at the edge router (OB), as well. Finally, the FCMDPF framework is evaluated based on the optimal specifications for a protective framework to protect web applications from all sorts of HTTP-based DoS and DDoS attacks, as are outlined above. The evaluation is unveiled in Table 2 above. It proved that the FCMDPF framework was successful in providing the full support to all the optimal specifications. In contrast, it suffers from low rate of false negatives, since it was not able to detect and prevent all of flash crowd (FC) attacks. As well, it failed to validate and trace back some of incoming requests. Future research will hopefully improve the accuracy rates of the FCMDPF framework.

## Figures and Tables

**Algorithm 1:**
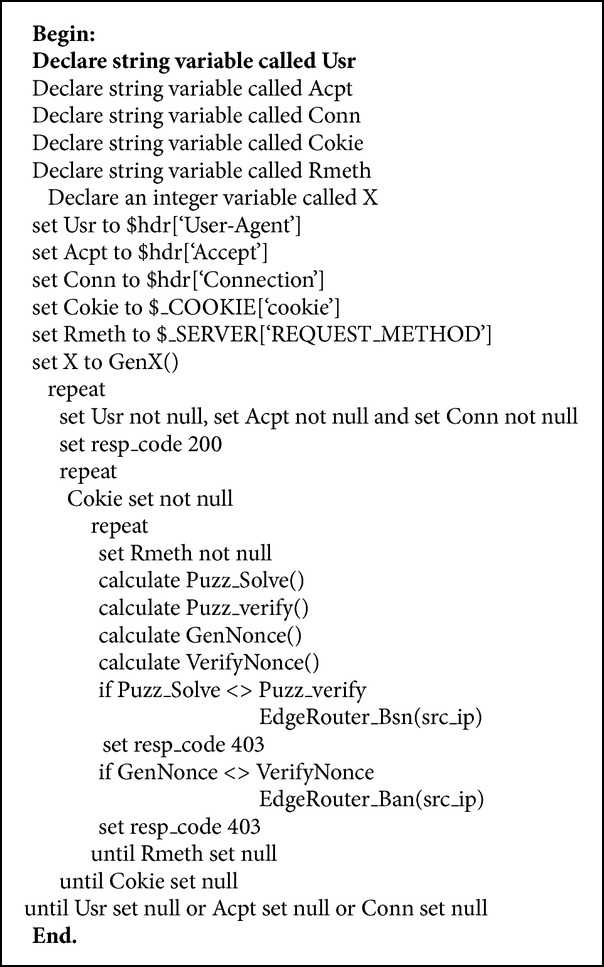
Service traceback oriented architecture (STBOA) algorithm.

**Algorithm 2:**
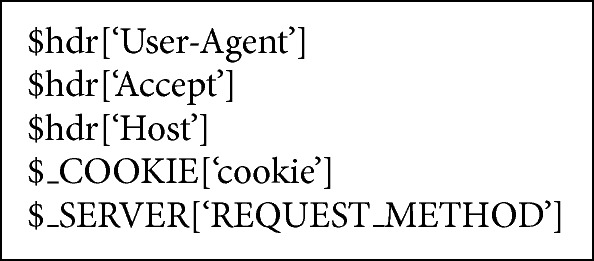
Request's headers checked by the STBOA algorithm.

**Algorithm 3:**
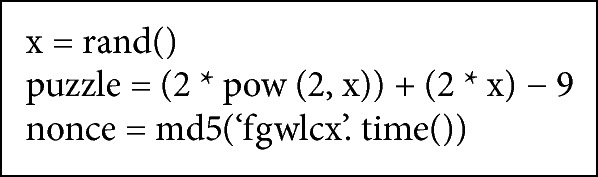
STBOA algorithm formulas to generate puzzle, random number, and nonce value.

**Algorithm 4:**
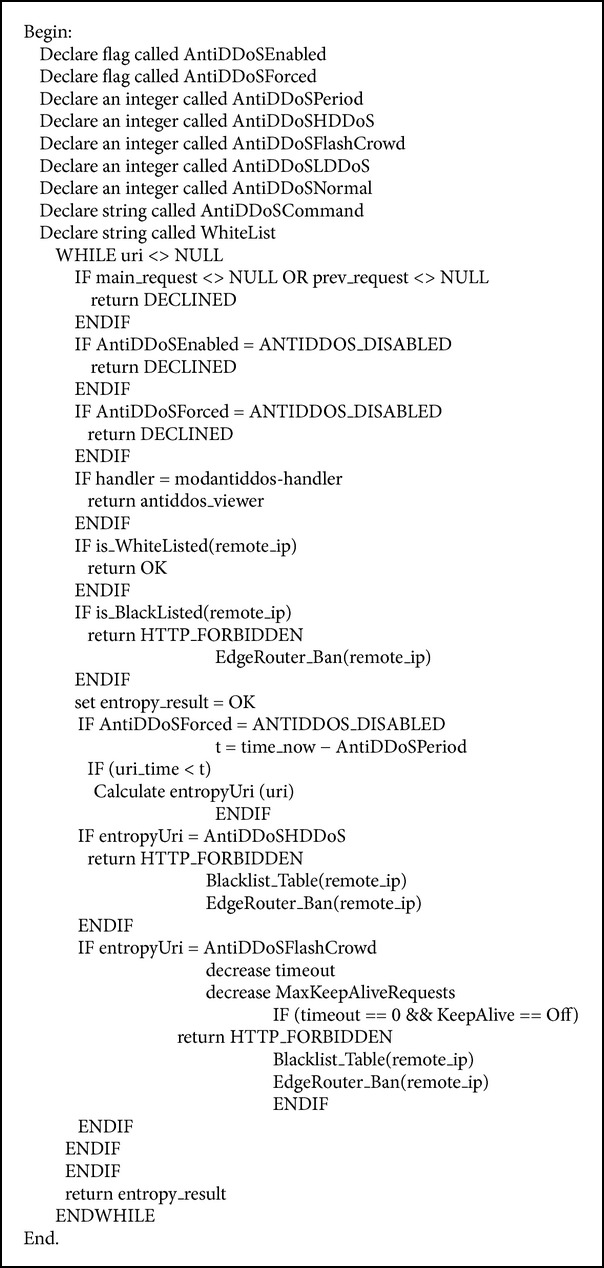
Flexible advanced entropy based (FAEB) algorithm.

**Algorithm 5:**
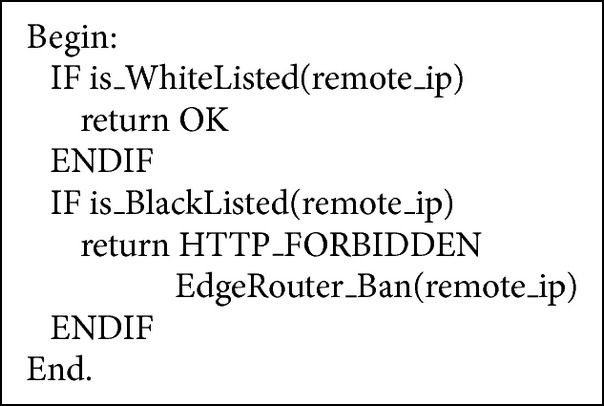
Whitelist and blacklist checking in the FAEB algorithm.

**Algorithm 6:**
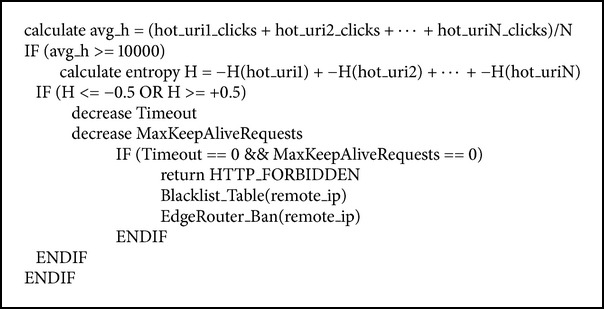
Flash crowd attack entropy algorithm.

**Algorithm 7:**
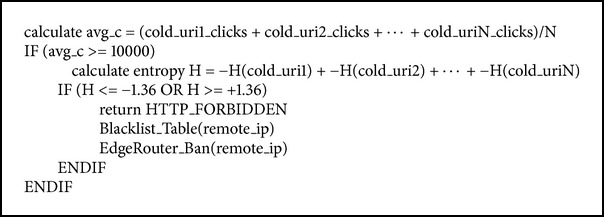
High rate attack entropy algorithm.

**Table 1:** Comprehensive survey on detective and preventive schemes and frameworks to all sorts of HTTP-based DoS And DDoS Attacks.

Number	Scheme/framework	Objective	Providing/protecting				
			HR-DDOS	LR-DDOS	FC	OB	TB & CV
1	Service oriented traceback architecture (SOTA) [1]	Identify attack source.	*✗*	*✗*	*✗*	*✗*	✓

2	Filtering tree [2]	Protect Cloud Computing against XML and HTTP DDoS attacks.	*✗*	*✗*	*✗*	✓	✓

3	Attack source identification at router level using marking algorithm [3]	Overcome IP spoofing.	*✗*	*✗*	*✗*	✓	✓

4	Confidence based filtering (CBF) [4]	Firewall web application.	✓	*✗*	*✗*	✓	*✗*

5	A New algorithm for detecting and defending CC attacks [5]	Protect web server from CC attacks.	*✗*	*✗*	*✗*	*✗*	✓

6	Intelligent decision prototype (IDP) [6]	Identify and defend attack source.	*✗*	*✗*	*✗*	✓	✓

7	Defense system for cloud computing [7]	Trace and identify the real source of DDoS attacks.	*✗*	*✗*	*✗*	✓	✓

8	EDoS-Shield [8]	Mitigate the economic denial of sustainability (EDoS) attack in the cloud computing.	*✗*	*✗*	*✗*	✓	✓

9	Diagnosis of network anomaly based on statistical traffic analysis [9]	Spot the anomalies of network Traffic.	✓	*✗*	*✗*	✓	*✗*

10	Dynamic hidden semi-Markov model (HTTP) [10]	Model the time varying user to detect web DDoS attacks.	✓	*✗*	*✗*	*✗*	*✗*

11	Enhanced fast-SCTF [11]	Detect and filter distributed reflection denial of service (DRDoS) attacks.	*✗*	*✗*	*✗*	✓	✓

12	IP to hop count mapping table (IP2HC) filtering technique [12]	Defend against IP spoofing attack.	*✗*	*✗*	*✗*	✓	✓

13	Transition matrix [13]	Detect HTTP application based DDoS attacks.	✓	*✗*	*✗*	*✗*	*✗*

14	Relative entropy based HTTP application DDoS detection [14]	Detect HTTP application based DDoS attacks.	✓	*✗*	*✗*	*✗*	*✗*

15	Analysis of network's traffic by using IP addresses correlation [15]	Detect network DDoS attacks.	✓	*✗*	*✗*	*✗*	*✗*

16	Large deviation measuring click ratio based web access behavior (LD-IID) scheme and large deviation measuring web access behavior based on Markov process (LD-MP) scheme [16]	Detect HTTP application based DDoS attacks.	✓	*✗*	*✗*	*✗*	*✗*

17	Chi-square based space (CSDM) Davison method [17]	Enhance anomaly detection system accuracy.	✓	*✗*	*✗*	*✗*	*✗*

18	An advanced entropy based DDoS detection scheme [18]	Determine the most suitable threshold value for detecting DDoS attacks accurately.	✓	✓	✓	*✗*	*✗*

19	HTTP reject [19]	Block user's requests on the IP layer during DDoS attacks and keep the end user to be notified as well.	✓	*✗*	*✗*	*✗*	*✗*

20	CALD [20]	Protect web server from flash crowd.	✓	*✗*	✓	✓	*✗*

21	VicSifter [21]	Detect DDoS attacks and determine the attack's victims at an early stage.	✓	*✗*	*✗*	*✗*	✓

22	Throttling DDoS attacks [22]	Eliminate and slow down the impact of DDoS attacks against web server.	*✗*	*✗*	*✗*	✓	✓

23	An early DoS/DDoS attacks detection method based on the concept of short-term entropy [23]	Focus on the early DoS/DDoS attacks detection.	✓	✓	*✗*	*✗*	*✗*

24	A real time DDoS attacks detection and prevention system based on the analysis of per IP traffic behavior [24]	Monitor and detect DDoS attacks near to the attack's source.	✓	*✗*	*✗*	✓	*✗*

**Table 2:** Evaluating FCMDPF framework based on the optimal specifications for a protective framework to protect web applications from all sorts of HTTP-based DoS And DDoS attacks.

Framework specifications/FCMDPF layers	OB layer	STBOA layer	FAEB layer	Remarks
(1) The framework should provide a protection against high rate DDoS (HR-DDoS) and flash crowd (FC) attacks, as well. It should be able to differentiate between them clearly to block the former immediately and block the latter gradually.	***✗***	***✗***	**✓**	The FAEB scheme of the FCMDPF framework is quite able to differentiate between HR-DDoS and FC attacks precisely. Hence, it is able to provide the protection for web applications against them properly through the FAEB algorithm, flash crowd attack entropy algorithm, and high rate attack entropy algorithm, as are described previously. The mod_antiddos module subsystem, which is developed based on FAEB scheme, detected and prevented all high rate HTTP-based DoS/DDoS (HR-DDoS) attacks. As well, it detected and prevented 369726 out of 420000 flash crowd (FC) attacks.

(2) The framework should provide a protection against low rate DDoS (LR-DDoS) attacks.	***✗***	***✗***	**✓**	Despite the FAEB scheme of the FCMDPF framework and therefore the mod_antiddos module subsystem, being able to protect web applications from LR-DDoS attacks, this protection is excluded intentionally in this research. It is excluded because the protection from LR-DDoS attacks is provided in all recent web servers by default.

(3) The framework should provide a mechanism to verify the validity of the incoming requests. As well, it should provide a mechanism to find out the true attacking IP source. Besides that, it should not be designed in a way that annoys the requesters by performing extra tasks, such as CAPTCHA.	**✓**	**✓**	***✗***	(i) The STBOA scheme of the FCMDPF framework and therefore STBOA_Shield subsystem, which is developed based on STBOA scheme, is quite able to verify the validity of an incoming request. The STBOA scheme verifies it through the STBOA algorithm to identify if it is legitimate or illegitimate and, therefore, subsequently to pass the former and block the latter. As well, it provides a mechanism that is quite able to trace back and find out the true attacking IP source in a way that does not burden or annoy the requester. In particular, the second phase of the STBOA scheme utilizes web services to send back a puzzle to the requester. In case the requester is a human using a real web browser (not a bot), he will answer this puzzle automatically by the browser itself without human interaction. Then, he will send back the answer to the web application. After that, the web application verifies (examines) the answer, and if it is correct, it passes it to the next layer or otherwise it blocks it immediately and updates the OB scheme blacklist database table with this attacking IP source. (ii) The STBOA_Shield subsystem, which is developed based on STBOA scheme, succeeded to validate and trace back 369726 out of 420000 incoming requests. (iii) The OB scheme then collaborates to block those updated attacking IP sources in upcoming incoming requests.

(4) The framework should provide a mechanism to block the attacking IP sources at the edge router near to the attacking source.	**✓**	***✗***	***✗***	(i) The outer blocking (OB) scheme of the FCMDPF framework is quite able to block the attacking IP source that neither passes the STBOA scheme's tests, nor passes the FAEB scheme's tests, at the Edge Router (Network Entrance). (ii) The OB_Shield subsystem succeeded to detect and prevent all of those attacking IP sources, which were 420000 IP sources, at the edge router.

(5) The framework should be designed in a way that supports the separation of duties concept.	**✓**	**✓**	**✓**	(i) The FCMDPF framework is a collaborative, multilayer, DDoS prevention framework because it protects web applications against HTTP DoS/DDoS attacks at the different collaborative points through which the incoming requests have gone. (ii) Each point at different framework layers collaborates to protect web applications from HTTP DoS/DDoS attacks by performing its special tests. Then, it forwards the request to the next framework's layer if it succeeds, or otherwise it will be dropped immediately. (iii) In the same manner, the next framework's layer performs its special tests, and then it forwards the packet to the next point if it succeeds, until it reaches the target. Otherwise, it will be dropped immediately.

(6) The framework should be compatible with the existing protocols.	**✓**	**✓**	**✓**	(i) The entire FCMDPF framework's layers, the OB layer, the STBOA layer, and the FAEB layer are compatible with existing protocols. (ii) Indeed, the OB layer is compatible with the IP, TCP, and UDP protocols. The OB layer merely uses the IP protocol to pass or block IP source the incoming request based on its blacklist database table. (iii) As well, the STBOA and FAEB layers are compatible with the HTTP protocol. (iv) The STBOA layer checks HTTP protocol headers and then generates a mathematical puzzle in order to verify the validity of the requester. After that, it passes it to the next layer if it is legitimate, or it blocks it immediately if it is illegitimate. (v) The FAEB layer uses the HTTP protocol's relevant information in order to detect HR-DDoS and FC attacks and to block the former immediately, while it blocks the latter gradually.

(7) The framework should be deigned explicitly for processing web application layer; HTTP protocol, rather than only network layer; IP and ICMP protocols, or transport layer; TCP and UDP protocols.	**✓**	**✓**	**✓**	The FCMDPF framework mainly concentrates on protecting the HTTP protocol from all sorts of DoS/DDoS attacks, such as HR-DDoS, LR-DDoS, and FC attacks. In addition, it traces back and finds out the true attacking IP sources.

(8) The framework should be easy to implement and does not cause huge processing and bandwidth overheads.	**✓**	**✓**	**✓**	(i) In reality, the FCMDPF framework is simple to implement through collaborative multilayer; each layer is distributed and deployed at different point. (ii) The FCMDPF framework generates very low processing and bandwidth overheads, compared to those schemes and frameworks that use packet marking [25].

(9) The framework should be able to adopt and update itself dynamically, once needed.	***✗***	**✓**	**✓**	The FCMDPF framework can adapt and update itself once needed. In particular, when a new stealthy bot's feature is discovered, the relevant feature's pattern can be easily added to the STBOA scheme. As well, when a new or a different profile is in need, the relevant information such as HR-DDoS and FC threshold's values can be easily added to the FAEB scheme.

(10) The framework should provide support to the hybrid scheme.	**✓**	**✓**	**✓**	In fact, the FCMDPF framework is designed in a way that supports the hybrid scheme that consists of proactive and reactive schemes. In particular, the OB and STBOA layers of the FCMDPF framework represent a proactive scheme, while the FAEB layer of the FCMDPF framework represents a reactive scheme.

(11) The framework should consume low storage memory.	**✓**	**✓**	**✓**	In general, the FCMDPF framework's layers, the OB layer, STBOA layer, and FAEB layer, consume very low memory storage. In particular, the OB layer of the FCMDPF framework consumes very low memory to store its blacklist database table, while the STBOA layer of the FCMDPF framework does not consume any storage memory, since all of its transactions are done in the real time. As well, the FAEB layer of the FCMDPF framework consumes little memory to store the relevant information about web pages.

(12) The framework should be resistant to IP source spoofing attacks, especially during finding out the true attacking IP sources.	***✗***	**✓**	***✗***	In fact, the FCMDPF framework is resistant to IP source spoofing attacks, since the STBOA scheme verifies whether the requester is legitimate or illegitimate by examining incoming request's headers and puzzle's answer. If the requester failed to satisfy these two tests, the requester is considered an attacker. Therefore, it will be blocked immediately.
